# Non-Steroidal Anti-Inflammatory Drugs in the Carcinogenesis of the Gastrointestinal Tract

**DOI:** 10.3390/ph3082495

**Published:** 2010-08-09

**Authors:** Debora Compare, Olga Nardone, Gerardo Nardone

**Affiliations:** Gastroenterology Unit, Department of Clinical and Experimental Medicine, University of Naples “Federico II”, Via S. Pansini 5, 80131 Naples, Italy; E-Mails: comparedebora@libero.it (D.C.); olga.nardone@libero.it (O.N.)

**Keywords:** cyclooxygenase-2, non-steroidal anti-inflammatory drugs, esophageal cancer, gastric cancer, colorectal cancer

## Abstract

It is estimated that underlying infections and inflammatory responses are linked to 15–20% of all deaths from cancer worldwide. Inflammation is a physiologic process in response to tissue damage resulting from microbial pathogen infection, chemical irritation, and/or wounding. Tissues injured throughout the recruitment of inflammatory cells such as macrophages and neutrophils, generate a great amount of growth factors, cytokines, and reactive oxygen and nitrogen species that may cause DNA damage that in turn predisposes to the transformation from chronic inflammation to neoplasia. Cyclooxygenase (COX), playing a key role in cell homeostasis, angiogenesis and tumourigenesis, may represent the link between inflammation and cancer. Currently COX is becoming a pharmacological target for cancer prevention and treatment.

## 1. Introduction

It was in 1863 that Rudolf Virchow indicated the link between cancer and inflammation on the basis of observations that tumours often arose at sites of chronic inflammation and that inflammatory cells were present in samples from tumours [1].

With time, many epidemiologic evidences have supported Virchow’s hypothesis showing that chronic inflammatory diseases are frequently associated with increased risk of cancers [1,2,3]. Currently it is estimated that underlying infections and inflammatory responses are linked to 15–20% of all deaths from cancer worldwide [4]. For instance, the risk of colorectal cancer (CRC) was 10-fold greater if linked with an inflammatory bowel disease, such as ulcerative colitis and Crohn’s disease [5,6]. The risk of esophageal, gastric and pancreatic cancer may be increased by inflammatory diseases, such as esophagitis with Barrett’s metaplasia, chronic atrophic gastritis with intestinal metaplasia and chronic pancreatitis respectively [7,8]. Furthermore, the cancer risk appears to be positively associated with the severity and duration of inflammatory diseases [9].

But the question now is how does chronic inflammation develop into tumours and which are the driving mediators in this process. Inflammation is a physiologic process in response to tissue damage resulting from microbial pathogen infection, chemical irritation, and/or wounding [2]. Tissues injured throughout the recruitment of inflammatory cells such as macrophages and neutrophils, generate a great amount of growth factors, cytokines, and reactive oxygen and nitrogen species that may cause DNA damage [3,10]. If the inflammatory process is activated persistently it may lead to continuous tissue damage and an altered microenvironment that in turn sustains cell proliferation and predisposes to the transformation from chronic inflammation to neoplasia [1].

The chronic inflammation microenvironment is predominated by macrophages [10]. Macrophages generate a great amount of inflammatory mediators which react with DNA and cause mutations in proliferating epithelial and stroma cells [11,12]. Macrophages and T lymphocytes may release tumour necrosis factor-α (TNF-α) and macrophage migration inhibitory factor to exacerbate DNA damage [13]. Macrophages, neutrophils, eosinophils, dendritic cells, mast cells, and lymphocytes are also found to be key components in the epithelial-originated tumours [14]. Indeed, inflammatory cells act as tumour promoters in inflammation associated cancers. Tumour-associated macrophages (TAM) are a major component of the infiltrate of most, if not all, tumours [15]. TAM derive from circulating monocytic precursors, and are directed into the tumour by chemoattractant cytokines called chemokines. Many tumour cells also produce cytokines called colony-stimulating factors that prolong survival of TAM. Furthermore, TAM also produce growth and angiogenic factors as well as protease enzymes which degrade the extracellular matrix. Hence, TAM can stimulate tumour-cell proliferation, promote angiogenesis, and favor invasion and metastasis [16,17]. Cytokines, including interleukins (IL), TNF-α, growth factors and colony-stimulating factors, are secreted or membrane-bound molecules that play a regulatory role in the growth, differentiation, and activation of immune cells [18]. Cytokine signaling could contribute to the progression of tumours in two aspects: the stimulation of cell growth and differentiation and the inhibition of apoptosis of altered cells at the inflammatory site [18]. Chemokines include the largest family of cytokines. Most tumours produce chemokines of the two major groups, α (or CCX) and β (or CC) [19,20]. In the inflammation process, chemokines, usually induced by cytokines, are major soluble regulators that control the directional migration of leukocytes to the inflammatory site. It is well established that chemokines are involved in the promotion of cancer. Moreover, chemokines also facilitate tumour invasion and metastasis in various cancer types and the balance between chemokines with proangiogenic and angiostatic activities is critical in regulating angiogenesis. Mechanistically, chemokines may contribute to tumour invasion and metastasis by mediating the directional migration of tumour cells to specific distal organs via circulation in a similar manner to its control of leukocyte migration [21,22,23,24].

Therefore, the relationship between cancer and inflammation is not simple but rather it is a complicated pathologic processes under the control of many driving forces. To address the details of development of inflammation- associated cancers, and further the transition from inflammation to cancer, it is necessary to investigate the specific roles of key regulatory molecules involved in this process. Here we centre the attention on cyclooxygenase (COX), which plays a key role in cell homeostasis, angiogenesis and tumourigenesis. The function of COX in linking inflammation to cancer is now becoming the target of intense investigation and starting to have implications for cancer prevention and treatment.

## 2. Cyclooxygenase-2

Cyclooxygenase is a rate-limiting enzyme in the synthesis of prostaglandins (PGs). It catalyses the conversion of arachidonic acid to PGG2, then to PGH2 which is subsequently converted to various physiologically active prostanoids, including PGE2, PGD2, PGF2a, PGI2 (prostacyclin) and thromboxane A2 (TXA2) by the relevant enzymes in a variety of cell types [25,26]. The COX enzyme exists in three isoforms, commonly referred to as COX-1, COX-2, and COX-3 [27,28]. COX-1 is expressed constitutively in many tissues and mediate the "housekeeping" functions such as cytoprotection of gastric mucosa, regulation of renal blood flow and platelet aggregation. In contrast, COX-2 is not detected in most normal tissues, but its expression may be induced mainly at sites of inflammation in response to inflammatory stimuli including pro-inflammatory cytokines such as IL-1α/β, interferon-γ (IFN-γ), TNF-α and growth factors [27]. COX-3, a novel COX-1 splice variant (now called COX-1b) has been identified in canine tissue (most abundant in cerebral cortex) as an acetaminophen-sensitive isoform. However, the implication of this splice variant in humans is still not known [28].

The stimulation of COX-2 expression in Src-transformed fibroblasts, endothelial cells and monocytes treated with the tumour promoter tetradecanoylphorbol acetate or lipopolysaccharide led to the notion that COX-2 is an inducible enzyme that produces prostaglandins during inflammatory and tumourigenic settings [29]. A review article on this issue showed that COX-2 is up-regulated from 51% up to 100% of the tumours by Northern blot, reverse transcription polymerase chain reaction, immunoblotting and immunohistochemistry [30].

The up-regulation of COX-2 results in an increased synthesis of PGs. Prostaglandins exert their effects locally in both autocrine and paracrine patterns. In particular PGE-related to COX-2 up-regulation appeared strongly involved in the carcinogenetic process. PGE2 effects are mediated by a family of G-protein-coupled receptors, namely, EP1, EP2, EP3, and EP4 [31]. In some cell types, nuclear peroxisome proliferator-activated receptors (PPAR) are also involved in mediating the PG effects [32]. In a recent study in CRC cells, PGE2 promoted cell growth and motility via the EP4 receptor by activation of the phosphatidylinositol 3-kinase (PI3K)/protein kinase B (Akt/PKB) pathway. Interestingly, EP2 and EP3 were also expressed in the CRC cells and their binding affinities to PGE2 are similar to EP4 [33].

The only other COX-2 derived PG implicated in oncogenesis is TxA2, which was reported to promote tumour growth and angiogenesis [34]. Moreover, it has been reported that TxA2 synthase inhibitor blocks colorectal carcinoma liver metastasis in an *in vitro* study [35]. COX-2 is involved in the carcinogenesis process throughout cellular proliferation, antiapoptotic activity, angiogenesis, and immune response.

### 2.1. Proliferation and apoptosis

Prostaglandins stimulate proliferation of different cell lines derived from gastrointestinal tract such as colonic, intestinal, gastric and esophageal cell lines. COX-2 derived PGE2 promotes human cancer cell growth by autoregulation of COX-2 expression, which depends primarily on PGE2 induced activation of the Ras-MAPK pathway [36].

Overall data from literature show that COX-2 inhibits apoptosis through three different pathways: the Bcl-2 mediated pathway, the nitric oxide pathway, and that of ceramide [37]. The role of COX-2 in preventing apoptosis is likely mediated by COX-2 derived PGE2, which attenuates cell death induced by the COX-2 selective inhibitor SC-58125 [38]. PGE2 induces antiapoptotic protein expression such as Bcl-2 and increases nuclear factor kappa B (NF-κB) transcriptional activity, which is a key antiapoptotic mediator [39].

COX-2-derived PGs regulate programmed cell death and reduce the apoptotic rate *via* inhibition of the mitochondrial apoptotic pathway characterized by reduced cytochrome C release, attenuated activation of caspase-9 and -3 and up-regulation of bcl-2 [36]. Additionally, increased prostanoid generation due to COX-2 overexpression specifically inhibits Fas-mediated apoptosis [40]. These findings have stimulated great interest in identifying COX-2 as a target for modulating apoptosis.

*In vivo*, both non-selective and selective COX-2 inhibitors stimulate apoptosis in APC-deficient cells that have not yet undergone malignant transformation. This is also seen clinically in familial adenomatous plyposis (FAP) patients treated with sulindac and in experimental studies of ApcMin mice and rats exposed to chemical carcinogens [41,42,43,44,45]. Non-selective COX-2 inhibitors lose their ability to inhibit chemically induced tumours when polyps undergo malignant transformation. In contrast, selective COX-2 inhibitors stimulate apoptosis and suppress growth in many carcinomas, including cultured human cancers of the stomach [46], esophagus [47], colon [48], and pancreas [49].

### 2.2. Angiogenesis

The formation of new blood vessels by angiogenesis to provide adequate blood supply is a key requirement for the growth of many tumours. While normal blood vessels express the COX-1 enzyme, new angiogenic endothelial cells express the inducible COX-2. Overexpression of COX-2 in CRC cells induces the production of angiogenic factors such as vascular endothelial growth factor (VEGF), basic fibroblast growth factor (bFGF), IL-8, TNFα, platelet derived growth factor (PDGF) and PGs. Several reports demonstrated that PGE2 up-regulates VEGF in cultured human fibroblasts and increases VEGF and bFGF expression through stimulation of ERK2/JNK1 signaling pathways in endothelial cells [50,51,52]. Interestingly, VEGF and bFGF induce COX-2 and subsequent PGs production in endothelial cells, suggesting that the effects of PGE2 on regulation of VEGF and bFGF are likely amplified through a positive feedback loop [53]. More recently, PGE2 has been shown to regulate angiogenesis through the modulation of a chemokine receptor signalling by modulating bFGF-induced chemokine receptor-4, that is important for microvessel assembly *in vivo*, and inducing the pro-angiogenic chemokine CXCL-1 *in* *vivo* [54]. PGE2 may also stimulate the transcription of the hypoxia inducible factor-1 (HIF-1) and work concomitantly with the hypoxic tumour microenvironment to orchestrate the process of angiogenesis [55].

The contribution of COX-2 at multiple points in the angiogenetic cascade makes it an ideal target in the pharmacological inhibition. Both non-selective and selective COX-2 inhibitors inhibit angiogenesis through a combined inhibition of angiogenic growth factors production, response to angiogenic factor and impairment of endothelial cell survival and migration. Inhibition of COX-2 activity in endothelial cells by COX-2 inhibitors resulted in a diminished integrine αVβ3-dependent activation Cdc42 and Rac, two members of the Rho family of GTPases that regulate cytoskeletal organization and cell migration, resulting in FGF-2-induced angiogenesis *in vivo* [56].

### 2.3. Immune response

The tumour microenvironment is predominantly shifted from a Th1 to a Th2 dominant immune response [57]. PGE2 has been shown to down-regulate Th1 cytokines (TNF-α, IFN-γ and IL-2) and up-regulate Th2 cytokines such as IL-4, IL-6 and IL-10 [58,59]. Moreover, PGE2 can modulate immune function through inhibiting dendritic cell differentiation and T cell proliferation and suppressing the antitumour activity of natural killer cells and macrophages [58]. In addition, PGE2 up-regulates the complement regulatory protein decay accelerating factor which results in blocking the complement C3 into two active compounds, C3a and C3b in CRC cells [60]. This ability of PGE2 to suppress these immune responses may allow tumour cells to escape immunosurveillance, adding to the already countless roles of the COX-2/PGE2 pathway during tumour development. COX-2 selective inhibitors restore the tumour induced imbalance between Th1 and Th2 and promote antineoplastic responses in lung cancer and metastatic spread of CRC [61,62]. These findings led to extensive efforts to understand how PGE2 can regulate immunosuppression.

### 2.4. COX inhibitors and cancer chemoprevention

Non-steroidal anti-inflammatory drugs (NSAIDs) block both COX-1 and COX-2 isoenzymes. In addition to the beneficial effects on the treatment of pain and inflammation, the use of NSAIDs is linked to other beneficial effects in the prevention and treatment of gastrointestinal tract tumours. The National Cancer Institute has tested many NSAIDs, such as ibuprofen, indomethacin, ketoprofen, piroxicam, and sulindac for chemopreventive activity [63]. However, the prolonged use of these compounds is limited by gastric (bleeding and ulcers) and kidney toxicity [64,65,66,67]. In addition, the use of steroidal antiinflammatories or glucocorticoids (e.g. dexamethasone) which can also inhibit COX-2 [68,69], is limited in the chemoprevention setting because of long-term adverse effects (adrenal cortical suppression). Recently, selective COX-2 inhibitors (valdecoxib, rofecoxib, celecoxib, and others yet in development) has been developed to minimize gastrointestinal toxicity because of the relative paucity of COX-2 expression in the gastrointestinal tract and the relative abundance of COX-2 expression in inflamed and painful tissues. However, selective inhibition of COX-2 might increase the risk for thrombotic cardiovascular events, due to a relative reduction in endothelial production of prostacyclin, while leaving the platelet production of TXA2 intact [70,71].

More long-term data are needed to fully evaluate the extent to which these important adverse side effects may be offset by other beneficial effects of NSAIDs and selective COX-2 inhibitors in cancer chemoprevention. 

### 2.5. Esophageal carcinogenesis

Esophageal adenocarcinoma (EAC) is generally considered to develop from gastroesophageal reflux disease through Barrett’s esophagus (BE). Over the past years, accumulating evidence has been obtained suggesting that increased COX-2 expression could be responsible for chronic inflammation related esophageal cancer promotion. Indeed, the incidence of COX-2 protein expression gradually increases with the development of esophageal lesions, from 75% in metaplasia, to 83% in low-grade dysplasia and up to 100% in high-grade dysplasia and EAC [72]. Shirvani *et al*. demonstrated that the expression of COX-2 increases parallel to the grade of dysplasia observed in BE [73]. Moreover, the same group demonstrated in an *ex vivo* model that both gastric acid and bile significantly elevated the expression of COX-2 [73]. Zhang *et al*. using a duodenogastroesophageal reflux model including unconjugated and conjugated bile acids, reported increased COX-2 expression in the esophageal mucosa followed by PGE2 production. Further, increased PG synthesis caused stimulation of cell proliferation and contributed to the development of dysplasia in Barrett's epithelium [74]. A recent metanalysis by Abnet *et al*. found a significant reduction in the incidence of esophageal cancer in aspirin or non-aspirin NSAID users (OR 0.64; 95% CI, 0.52–0.79 and OR 0.65; 95% CI, 0.50–0.85 respectively) [75].

Experimental models have demonstrated reduced expression of an apoptosis ligand, *Fas* (CD-95), in esophageal dysplastic and malignant tissues [76]. This reduced expression is linked to overexpression of COX-2, which in turn down-regulates the expression of *Fas* ligand [40]. A mechanism for the inhibitory effects of aspirin and NSAIDs on the occurrence of EAC could be the induction of apoptosis by COX-2 inhibition. Overexpression of COX-2 also was associated with increased levels of *bcl*-2, a proapoptotic protein that induced resistance to apoptosis [77]. Therefore, selective COX-2 inhibitors may up-regulate the expression of *Fas* receptors on the cell surface in subjects with Barrett dysplasia and have an inhibiting role in esophageal carcinogenesis by influencing apoptosis and cellular proliferation. Finally, COX-2 expression might be a prognostic marker in patients with Barrett's adenocarcinoma, as expression of COX-2 correlates with patient survival. Further support for the role of COX-2 derived PGs in the carcinogenesis emerged from the animal study by Buttar *et al*. in which both non-selective COX inhibitor (sulindac) and selective COX-2 blocker (MF tricyclic) significantly attenuated the incidence of Barrett adenocarcinoma [78]. Moreover, Kaur *et al*. studied the effect of COX-2 inhibitor (rofecoxib) on the marker of cell proliferation PCNA. In BE the authors found a significant increased PCNA expression as compared to normal esophageal mucosa. In addition, therapy with rofecoxib caused a significant inhibition of cell proliferation as evidenced by the decreased PCNA expression. Rofecoxib therapy led also to significant down-regulation of COX-2 expression in the Barrett's epithelium [79]. The suppressive effects of a COX-2 inhibitor, NS398, on the epithelium of BE have been demonstrated in two independent *in vitro* studies [80,81]. An increase in apoptosis and a suppression of cell proliferation are supposed to be responsible for the inhibition of cancer cells. Furthermore, in a study carried out using a carcinogen-induced rodent model, selective COX-2 inhibitors have been reported to prevent the development of esophageal cancer. *N*-nitrosomethylbenzylamine-induced esophageal tumourigenesis in rats was prevented by the administration of another selective COX-2 inhibitor, JTE-522 [82]. Nevertheless, a Chemoprevention Barrett's Esophagus Trial (CBET) started in 2003 as a phase IIb, multicenter, randomized, double-masked, placebo-controlled study of celecoxib in patients with Barrett's dysplasia failed to prevent progression of Barrett’s dysplasia to cancer. However, the apparent inability of celecoxib, compared with placebo, to decrease the percentage of samples with dysplasia is probably due to the several limitations of the study (previous diagnosis of displasia but no evidence of displasia at enrollment, imperfect biopsy sampling, natural reversion of dysplasia without any intervention, inadequate utilization of dysplasia grading as predictor of cancer because of the low intra- and interobserver agreement among pathologists) [83].

### 2.6. Gastric carcinogenesis

Gastric cancer (GC) is one of the most frequent malignancies worldwide [84]. The development of GC, at least of intestinal type, occurs on the basis of atrophy-metaplasia-dysplasia-sequence [85]. This multistep process is triggered by *Helicobacter pylori* (*H. pylori*) infection [85]. Indeed, the colonization of gastric mucosa with this bacterium causes a chronic inflammatory reaction with increased production of proinflammatory cytokines and generation of reactive oxygen species [86]. Interestingly, the presence of *H. pylori* also correlates with an up-regulation of the expression of COX-2 mRNA and PGE2 in GC cell lines [87].

Normal gastric mucosa scarcely expresses COX-2, but the expression of COX-2 and the production of eicosanoids (especially PGE2) increases through the multistep process of gastric carcinogenesis [88,89]. Ristimaki *et al*. described for the first time in 1997 an elevated expression of COX-2 in GC [90]. Since then, numerous studies have reported the relationship between COX-2 expression and gastric carcinogenesis. Sun *et al*. by immunohistochemistry reported a progressive positive rate of COX-2 from superficial gastritis, to gastric atrophy, intestinal metaplasia, dysplasia, and cancer (10.0%, 35.7%, 37.8%, 41.7%, and 69.5%, respectively) [91]. The COX-2 expression is more frequent in intestinal type than in diffuse type GC [92,93], and it also correlates with tumour size, depth of invasion, lymph node metastasis, lymphatic invasion, clinical stage, and prognosis [94,95,96,97,98]. This suggests that COX-2 expression may be an early event in gastric carcinogenesis process even if, the precise mechanisms leading to the overexpression of COX-2 are still not fully understood. However, there is evidence that proinflammatory cytokines and different gastric mucosal growth factors such as transforming growth factor alpa (TGFα) or hepatocyte growth factor (HGF) or finally gastrin could be involved in this process [99].

Previous studies demonstrated an increased gastrin level in the GC tissue. Gastrin is a potent stimulator of HGF expression and possesses also anti-apoptotic capabilities by inducing the antiapoptotic- proteins Bcl-2 and surviving [100,101]. The importance of gastrin and its precursor progastrin in mediating of COX-2 dependent gastric carcinogenesis was demonstrated in humans with GC treated with COX-2 inhibitor rofecoxib [102]. Treatment of GC patients with rofecoxib (50 mg/day) resulted in a significant decrease in plasma and tumour contents of both progastrin and gastrin levels, and this was accompanied by the increased expression of proapoptptic proteins such as Bax and caspase-3 with a concomitant reduction in Bcl-2 and survivin expression. The blockade of COX-2 was also associated with a decrease in the serum level of proinflammatory cytokines IL-8 and TNFα being also involved in the gastric carcinogenesis [103].

Experimental evidence has shown that COX-2 influences key cellular events, including apoptosis, cell cycle control, cell proliferation, and angiogenesis [50,77,104,105,106]. Selective COX-2 inhibitors (NS-398 and JTE-522), indomethacin, and aspirin can suppress cell replication, induce apoptosis, and reduce epidermal growth factor in gastric carcinoma cell lines (KATO III) [107,108,109]. Nam *et al*. examined the effect of nimesulide on gastric carcinogenesis using an *N*-methyl-*N*-nitrosourea (NMU)-induced and an *H pylori*-infected mouse model, demonstrating that gastric tumours developed in 68.8% of mice given both MNU and *H pylori*, whereas the tumour incidence in the mice receiving nimesulide in addition to MNU and *H pylori* was 27.8% [110]. More recently COX-2 was proven to have a strong relationship with gastric tumourigenesis in a study using transgenic mice. In the transgenic model expressing both COX-2 and microsomal prostaglandin E synthase (mPGES)-1, the animals developed inflammation- associated hyperplastic gastric tumours in the proximal glandular stomach [111]. In addition, NS-398 treatment for four weeks completely suppressed the gastric hypertrophy, thereby reducing the mucosal thickness in the same model [112].

Epidemiologic studies also have shown a decreased frequency of GC in people who take NSAIDs, Several case-control and cohort studies on NSAIDs use in gastric carcinoma have demonstrated a chemopreventive effect of NSAIDs [113,114,115]. Coogan *et al*. found that regular NSAID use (at least 4 days a week for >3 months) reduced the risk of GC in a hospital-based-case-control study of 254 patients (OR 0.3; 95% CI, 0.1–0.6). The protective effect was more pronounced among those patients using NSAIDs continually for >5 years (OR 0.2; 95% CI, 0.1–0.7) than for those using NSAIDs for <5 years (OR 0.4; 95% CI, 0.1–0.9) [114]. In a large cohort study of 635,031 subjects followed over 6 years, the American Cancer Society demonstrated that regular exposure to aspirin (>16 times/month) exerted a protective effect against GC; aspirin users were found to have approximately 50% the risk of GC compared with nonusers (OR = 0.53; 95% CI, 0.34–0.81) [93]. A recent metanalysis by Abnet *et al*. found a significant reduction in the incidence of GC in aspirin or non-aspirin NSAID users (OR 0.74; 95% CI, 0.64–0.87 and OR 0.79; 95% CI, 0.71–0.89 respectively) [75].

This evidence suggests that inhibition of COX-2 may be an attractive target for treatment and prevention of GC. However, upper gastro intestinal bleeding is a common side-effect of aspirin therapy, so co-administration of aspirin and proton-pump inhibitors is an attractive option in this setting, and is currently being studied in the AspECT study of esomeprazole and aspirin in patients with Barrett’s esophagus [116].

### 2.7. Colorectal carcinogenesis

Colorectal cancer is one of the most popular cancers in westernized countries [117]. CRC develops in a stepwise manner from aberrant crypts to adenomas, with increasing grade of dysplasia and finally to cancer. According to this adenoma-carcinoma sequence model, carcinogenesis proceeds through the accumulation of series of epigenetic and genetic alterations involving several tumour-suppressor genes *(i.e*., *APC and p53)* and oncogenes *(i.e*., *k-ras)* [118,119]. Among these COX-2 oncogene has been most intensively elucidated in both basic and clinical research due to its pathogenetic implication.

In normal human epithelium, COX-2 generally is down-regulated and is not expressed in the gastrointestinal tract. Dubois *et al*. were the first to report increased expression of COX-2 in CRC [120]. Their original observation was followed by several reports that confirmed increased COX-2 expression in this setting. An epoch-making paper was published by Oshima *et al*. in 1996 about the contribution of COX-2 to carcinogenic sequence in Wnt/Apc/Tcf pathway. They induced COX-2 mutations in *ApcΔ716* knockout mice, which led to the development of numerous polyps in the intestine. In COX-2−/− *ApcΔ716* and COX-2+/− *ApcΔ716* mice, the number of polyps dramatically decreased by 86% and 66%, in comparison to that in the littermate COX-2+/+ *ApcΔ716* mice [121]. 

Many studies have demonstrated that COX-2 is expressed early during the adenoma-carcinoma sequence, suggesting COX-2 should be in first line linked to the colorectal carcinogenesis. COX-2 expression is up-regulated by approximately 50% in colorectal adenoma [122] and 85–90% in CRC [105]. COX-2 overexpression appears to be associated with both the histological type and the location of the tumours. Overexpression was less prominent in tumours with signet cell morphology and was found more frequently in rectal carcinoma compared to carcinoma at others sites in the colon [123,124]. Furthermore the overexpression of COX-2 in CRCs appears to be associated with the genetic and epigenetic make-up of the tumours being significantly lower in proximal carcinomas that has the micro satellite instability (MSI) phenotype [123].

COX-2 is also expressed in the stromal compartment of adenomatous polyps and of invasive carcinomas both in experimental animal models and in humans. These stromal cells have the morphological and immunohistochemical characteristics of inflammatory cells [125,126,127].

Transfection of human CRC cells with a COX-2 expression vector resulted in increased invasiveness and activation of matrix metalloproteinases compared to the parental cell line. COX-2 overexpressing cells also produced proangiogenic factors, stimulated endothelial migration and tube formation and produced the proangiogenic factor VEGF [128].

In chemically (1,2-dimethyldralazine -DMH- and azoxymethane -AOM-), induced CRC in rat, the inhibition of PGs by non-selective NSAIDs and selective COX-2 inhibitors significantly reduced formation of aberrant crypts and development of adenomas and CRC [129]. Moreover, coxibs (rofecoxib) and non-selective NSAID (sulindac) significantly reduced the number and size of intestinal polyps in the mice with dysfunctional APC gene (APCD716 mice) [130]. Jacoby *et al.* by using the *Min* mice model showed that celecoxib decreased not only tumour size but also caused a decrease in the size of established polyps in the regression study [131].

In human CRC cell lines, HCA-7, which express high levels of COX-2 protein constitutively, and HCT-116 cells, which lack COX-2 protein, studies were conducted to investigate the relationship between inhibition of intestinal cancer growth and selective inhibition of the COX-2 pathway. Treatment of nude mice implanted with HCA-7 cells with a selective COX-2 inhibitor (SC-58125) reduced tumour formation by 85–90%. Colony formation of cultured HCA-7 cells also was inhibited by SC-58125. On the other hand, SC-58125 had no effect on HCT-116 implants in nude mice or colony formation in culture [132]. In addition Chan *et al*. found that regular use of aspirin appears to reduce the risk of CRCs that overexpress COX-2 (RR 0.64; 95% CI, 0.52–0.78) but not the risk of CRCs with weak or absent expression of COX-2 (RR 0.94; 95% CI, 0.73–1.26) [133]. This evidence suggests that a correlation may exist between inhibition of CRC cell growth and selective inhibition of the COX-2 enzyme.

In a National Cancer Institute-sponsored double-blind, placebo-controlled trial, celecoxib helped to reduce the number of colon polyps that occurred in patients with FAP. In this study, 77 patients were randomly assigned to treatment with celecoxib (100 or 400 mg twice/day) or placebo for 6 months. After 6 months, the patients receiving celecoxib 400 mg twice/day had a 28.0% reduction in the mean number of colorectal polyps (p = 0.003) and a 30.7% reduction in the polyp size (p = 0.001), as compared with reductions of 4.5% and 4.9%, respectively, in the placebo group. The occurrence of adverse events was similar among the groups [134]. The results of the study led to the approval of celecoxib by the United States Food and Drug Administration (FDA) as an adjunct to usual care for patients with FAP. In, another placebo-controlled study, rofecoxib given daily at a dose 25 mg, significant decreased the size and number of rectal polyposis in patients with FAP after 9 months [135]. Finally, Phillips *et al*. showed a significant reduction in duodenal polyps in patients with FAP treated with selective COX-2 inhibitor [136].

The data obtained from FAP patients encouraged the conduction of further studies in patients with sporadic adenomas and CRC. Baron *et al*. investigated the adenoma recurrence in 1121 patients with a history of sporadic colorectal adenomas randomized to receive placebo (n = 372), 81 mg aspirin (n = 377) or 325 mg of aspirin (n = 375) daily. Relative risks for advanced lesions were 0.59 (0.38–0.92) in the 81 mg group and 0.83 (0.55–1.23) in 325 mg group as compared to placebo. Surprisingly, the lower aspirin dose had stronger chemopreventive effect that the higher one. However, the assessment of possible chemopreventive effect of aspirin on colorectal carcinogenesis was limited by the short follow-up time of the study [137]. A recent prospective cohort study involving 1,279 subjects (549 who regularly used aspirin, 730 who did not use aspirin) with a diagnosed CRC, followed up to 12 years has shown a lower risk of CRC specific and overall mortality in aspirin users vs non-users (HR 0.71; 95% CI, 0.53–0.95). [138]. The Approve trial, a randomized multicenter, placebo controlled, double blind trial to investigate whether the chronic use of the coxib (rofecoxib 25 mg daily) would reduce the adenoma recurrence in 2,586 patients with a history of colorectal adenomas. Therapy with rofecoxib was associated with a significant reduction in adenoma number and size. Unfortunately, an increase in rofecoxib associated cardiovascular adverse events beginning at 18 months was also noted, which led to early study termination [139]. Similarly, Bertagnolli *et al*. in a five-years efficacy and safety analysis of the adenoma prevention with celecoxib trial, found an inhibitory effect of celecoxib in colorectal adenoma formation but they reported an elevated risk for cardiovascular and thrombotic adverse events [6% (RR, 1.6; 95% CI, 1.0–2.5) and 7.5% (RR, 1.9; 95% CI, 1.2–3.1) in celecoxib 200 and 400 mg twice daily users, respectively compared to 3.8% in placebo group [140].

The role of COX-2 inhibitors has been investigated in the treatment of advanced human CRC. The 14-day therapy with celecoxib (200 mg/day) caused a significant decrease in the progastrin and gastrin levels in the CRC tissue as well as significant decrease in the survivin expression [141]. Based upon these results it has been hypothesized that celecoxib therapy could contribute to the treatment of CRC *via* suppression of the anti-apoptotic proteins and reduction in progastrin-promoted tumour growth.

Since the overexpression of COX-2 in tumour may counteract the efficacy of cytotoxic chemotherapy due to the apoptosis resistance, the combination of chemotherapy with coxibs seems to be an attractive strategy to enhance the antitumour activity. Until now, the number of clinical studies in which rofecoxib was administered with chemotherapy in patients with CRC is very limited. Beccera *et al*. reported a phase II study in which rofecoxib was administered in combination with 5-fluorouracil and leucovorin in patients with metastatic CRC. The study was terminated when it was noted an increased toxicity (upper gastrointestinal bleeding, stomatitis, thrombocytopenia, diarrhea) in patients treated with chemotherapy and rofecoxib [142]. The addition of COX-2 inhibitor to the chemotherapy did not increase the efficacy of the antitumour activity of the chemotherapy. Despite these disappointing results, further studies with chemotherapy and COX-2 inhibitors will be needed to determine whether specific COX-2 therapy is able to improve patient outcome with a reasonable safety profile.

Finally, there are some groups postulating that both COX-isoforms are involved in the intestinal tumourigenesis. Chulada *et al*. demonstrated that deficiency of either COX-1 and COX-2 caused similar reduction in intestinal tumourigenesis in *Min* mice having a mutation in the APC gene and spontaneously developing intestinal adenomas . Furthermore, both COX-isoforms contributed to PGE2 production in polyps [143]. Finally, the inhibitory effect of non-selective NSAIDs and coxibs was demonstrated in xenograft mice models in which CRC cell lines are injected and form tumours with metastasis [62]. 

## 3. Conclusions

Mounting evidence gained from studies with cancer cell lines, mouse models and a number of clinical trials with both non-selective and selective COX-2 inhibitors support the notion of an important role for the COX-PG pathway in the development of gastrointestinal tumours. However, the mechanisms of the anti-tumoural action of the COX-2 inhibitors still remain to be defined and may vary from agent to agent and tumour to tumour.

*In vitro* studies have shown a mixture of COX-related mechanisms in controlling proliferation and apoptosis balance. Animal model studies are often performed with much higher pharmacological doses than those clinically achievable. Human observational studies are prevalently of the case-control type and often suffer from inadequate sample size to avoid a type II statistical error. In addition, more studies are needed to define the lowest effective dose, the age at which to initiate therapy, the optimum treatment duration and the subpopulations for which the benefits of chemoprevention outweigh the risks of adverse side effects. Furthermore, due to the high cost of these new agents, cost-effectiveness analyses must be carried out to optimize the allocation of resources. The cumulative probability of developing a lesion from birth to 80 years of age is less than 4% thus, in the general population, more than 95% of people treated prophylactically with COX-2 inhibitors will not benefit.

Understanding the molecular mechanisms of COX-2 and its downstream targets will help to identify specific molecular targets for developing new drugs which target this pathway, however, until now inconsistency still exists regarding the specific role of COX-2 in linking inflammation and cancer. 

## 4. Future Directions

There are many reasons to be optimistic that tumour formation mediated by abnormal COX-2 activity can be effectively and safely targeted by chemoprevention regimens. Available anti-COX-2 agents vary substantially in their activities, with different degrees of COX-1 *versus* COX-2 selectivity, differences in antitumour efficacy, and differences in toxic effects. In the balance of factors that maintain control over the highly reactive prostaglandin system, small differences in agent selectivity, dose, drug metabolism, and patient reactivity or predisposition to toxic effects could be important. Presently, we have little information about the relation between these important variables and agent efficacy and toxic effects. The rapid development of safe and effective inhibitors targeting individual COX enzymes could not only dramatically improve our understanding of the function of COX-2, but also result in discovery of COX independent functions of NSAIDs, providing important hints for future drug design. 

An exciting, novel concept in cancer chemoprevention may be the use of combination therapy, which may allow dose reduction (and hence decreased systemic bioavailability) of NSAIDs or coxibs when combined with other anti-cancer agents, e.g., epidermal growth factor receptor inhibitors. Alternatively, other steps in PG biosynthesis and signalling represent potential targets. Indeed, pharmacological inhibitors of PGE2-EP receptors, which have anti-neoplastic activity, have been generated. Development of specific inhibitors for individual enzymes and receptors will be dependent on better understanding of the roles of particular PGs and their signaling receptors in health and disease. Finally, inducible expression of COX-2 is tightly controlled at the transcriptional and translational level in a cell-specific manner. Therefore, targeting mechanisms controlling neoplastic COX-2 regulation in stromal and epithelial elements of tumours may provide an alternative in tumour-specific COX-2 inhibition, avoiding the side effects related to systemic COX-2 inhibition.

In the light of an innovative alternative to these pharmacological approaches, it has been proposed the possible strategy to achieve a strong and selective inhibition of COX-2 enzyme by using the mechanism of RNA Interference targeted against its mRNA. Anti-COX-2 RNA molecules can be generated in CRC cells from short hairpin RNA precursors, delivered *in vitro* by a retroviral expression system, and induce a significant and stable silencing of overexpressed COX-2 in human CRC cells. As a safer alternative to viral approach, nonpathogenic bacteria (*E. coli*) can be engineered to invade eukaryotic cells. Moreover, the involvement of micro-RNAs in COX-2 posttranscriptional regulation opens up the possibility to exploit an endogenous silencing mechanism to knockdown overexpressed COX-2. Thus, these recent strategies disclose new challenging perspectives for selectively inhibiting COX-2 enzyme.
